# Guidelines unmet: Assessing gaps in breast cancer survivorship care

**DOI:** 10.1177/17455057241306809

**Published:** 2024-12-13

**Authors:** Dana Pearl, Anna N. Wilkinson

**Affiliations:** Department of Family Medicine, University of Ottawa, Ottawa, ON, Canada

**Keywords:** breast cancer, survivorship, primary care, survivorship care plan

## Abstract

**Background:**

In Canada, after completing their treatment at oncology centers in tertiary care facilities, most breast cancer patients are discharged and receive survivorship care from primary care providers (PCPs). Evidence-based guidelines exist to inform appropriate care for breast cancer survivor follow-up.

**Objectives:**

This study analyzed the concordance of breast cancer survivorship follow-up care by PCPs with recommended guidelines at an academic Family Health Team (FHT) in Ottawa.

**Design:**

Retrospective chart review of electronic medical records of rostered patients from FHT.

**Methods:**

Data was extracted from the charts of 60 breast cancer survivors. Concordance of breast cancer survivorship care by PCPs with evidence-based guidelines was established in three key survivorship domains: surveillance for recurrence or new cancers, management of treatment side effects and preventative health.

**Results:**

PCPs provide care concordant with guidelines only 20% of the time, with areas such as preventative care at 86.7% concordance far better than management of side effects at 58.3% and oncological surveillance at 38.3%. Care did not significantly differ by age at diagnosis.

**Conclusion:**

These results highlight gaps in the current survivorship care delivery and function as a baseline for comparative analyses for future interventions to optimize survivorship follow-up care.

## Plain language summary

Beyond treatment: How well are we caring for Breast Cancer Survivors?

Are breast cancer survivors followed by family doctors getting appropriate care?

**Why was this study done?:**

Following treatment for breast cancer in Canada, most patients move out of the cancer care system and receive their survivorship care from primary care providers (PCPs). Although specific guidelines exist to assist PCPs in caring for breast cancer survivors, it remains unclear how closely their practices align with these recommendations. This study aims to evaluate the adherence of PCPs to the established guidelines for breast cancer survivorship care.

**What did the researchers do?:**

The research team reviewed the charts of breast cancer survivors in a large urban academic family health team. They looked specifically at how well the family doctors were following the guidelines in three categories: looking for new or recurrent cancers, managing the side effects of the breast cancer and its treatments, and preventative care.

**What did the researchers find?:**

The results showed that adherence to the recommended guidelines for breast cancer survivorship care was only 20%. However, there were significant variations across different areas of care: preventative health services aligned with guidelines 86.7% of the time, management of treatment side effects was at 58.3%, and oncological surveillance was the lowest at 38.3%. The quality of care provided did not significantly differ based on the patients’ age at diagnosis.

**What do these findings mean?:**

This study has identified important gaps in the survivorship care provided to breast cancer patients, underscoring the need for improvements in follow-up processes. By highlighting shortcomings in survivorship care, this study establishes a baseline for future research and interventions aimed at improving care for breast cancer survivors. Better adherence to guidelines can lead to improved health outcomes and stronger support for those adjusting to life after cancer treatment.

## Introduction

Breast cancer is the most common type of cancer and the second most common cause of cancer-related death affecting women in Canada. In Canada in 2022, 28,600 women were diagnosed with breast cancer and 5500 women died of breast cancer. With the advent of better treatments, such as targeted and endocrine therapy, and robust provincial breast cancer screening programs, it is estimated that 89% of women with breast cancer are survivors at 5 years post-diagnosis.^1^

The initial post-treatment years are critical not only for cancer surveillance but also for addressing the health-related and psychosocial aftermath of cancer and its treatment.^2^ As the number of breast cancer survivors in Canada continues to grow, it is primary care providers (PCPs) who care for these patients.^3^ PCPs are well situated to provide follow-up care to breast cancer survivors as they specialize in longitudinal and prevention-focused healthcare and can provide quality care on par with medical oncologists and tertiary specialist centers at a lower cost to the system.^4^ However, there are several documented barriers which prevent PCPs from providing optimal multidimensional survivorship care including knowledge of the guidelines, sense of inadequate preparation, lack of time and resources and scarcity of experience.^5,6^

Survivorship care after breast cancer includes three domains: surveillance for recurrent and new cancers; managing the side effects and consequences of cancer and its treatment, including physical and psychosocial outcomes; and preventative care.^7^ These domains and specific recommendations for evidence-based care maneuvers are described in the Breast Cancer Survivorship Tool, a peer-reviewed tool which provides national guidance for Canadian family physicians.^8^ (Table 1). In this project, we analyze the concordance of follow-up care of breast cancer survivors by PCPs after discharge from the cancer center with the evidence-based recommendations in the Breast Cancer Survivorship Tool. This study provides important insights into PCP care practices for breast cancer survivors and identification of potential gaps in the management of these patients.

**Table 1.** Evidence-based recommended survivorship care summarized from breast cancer survivorship tool.^8^

Cancer surveillance	Medical follow-up appointments: focused history and clinical breast/chest wall exam every 6–12 months for the first 1–3 years, and then annually from year 4 onwards
	Surveillance mammography (unless bilateral mastectomy or breast reconstruction with implants) on an annual basis
	Surveillance MRI (in addition to mammography) on annual basis for high-risk women (as determined by the Ontario Breast Screening Program)
	Breast self-examinations on monthly basis
Preventative care	Routine screening for cervical cancer (Pap tests every 3 years)
	Routine screening for colon cancer (FIT every 2 years or colonoscopy every 5–10 years)
Management of side effects	Screening DEXA scans to determine BMD every 2 years for patients on aromatase inhibitors, pre-menopausal women taking tamoxifen of GnRH agonist or chemotherapy-induced premature menopause
	Assessment of psychological distress, anxiety, and depression on (at least) an annual basis
	Monitor lipids annually if patients are on aromatase inhibitors

DEXA: dual-energy-X-ray absorptiometry; BMD: bone mineral density; GnRH: gonadotropin-releasing hormone; MRI: magnetic resonance imaging; FIT: fecal immunohistochemistry testing.

## Methods

A retrospective chart review was performed for breast cancer survivors who were patients of the Ottawa Hospital Academic Family Health Team (FHT), a large, multi-site, urban academic teaching family practice in Ottawa, Ontario, with approximately 17,000 patients, associated with The Ottawa Hospital and the University of Ottawa. Patients had to meet the following inclusion criteria: female patients, age over 18, diagnosed with breast cancer in 2015 or later (to include only survivorship care in the first 5 years after treatment completion), no metastatic disease, treated by oncology in Ottawa, discharged from oncology to PCP survivorship care after a single discharge visit at the Wellness Beyond Cancer (WBC) Program, and care plan present in the electronic medical record (EMR). Exclusion criteria included: diagnosis of breast cancer prior to 2015, patients that were not yet discharged from oncology at the tertiary cancer center, lack of WBC care plan (discharge document), data not fully accessible in EMR and discharge by WBC within the last 6 months (before PCP follow-up requirements are due). This study was completed with data with ranging from when our EMR (EPIC) was launched in 2019 until October 2023.

Data extraction from the EMR included: current age, year of breast cancer diagnosis, age at diagnosis, and type(s) of treatment received (surgery, radiation, targeted therapy, chemotherapy, and/or endocrine therapy). Documented discharge from oncology was noted, as well as the presence of a WBC Program care plan in the EMR.

Concordance with recommended care was determined by extracting completion of annual mammograms, clinical breast/chest wall exams, assessment of distress, and anxiety and/or depression. For patients taking aromatase inhibitors, the completion of bone mineral density (BMD) and lipids were also noted. The performance of routine cancer screening investigations including cervical screening with Pap tests and colon screening with either fecal immunohistochemistry testing (FIT) or colonoscopy as indicated was noted.

The reporting in this study conforms to the SQUIRE 2.0 framework for quality improvement in health care.^9^

### Statistical analysis

The results were analyzed using descriptive analysis tools, including percentage of patients with completed follow-up items across all modalities, and measures of central tendency and variability. The patients were also grouped into categories based on age at the time of diagnosis, <50, 50–59, 60–69, and ⩾70, to determine a correlation between age and concordance of follow-up care recommendations with evidence-based guidelines. The results were also stratified based on the three domains of follow-up care. Two-sided chi-square tests, with a significance level of 0.05, were used to test the null hypothesis of no difference in proportions.

## Results

The FHT had 211 breast cancer survivors who were rostered patients. Of the 211, 60 patients met the inclusion criteria and were eligible for chart review. The average year of diagnosis of breast cancer was 2018 (standard deviation (SD) = 2.2). The average age at diagnosis was 62.7 years old (SD = 10.8), with the youngest patient diagnosed at age 39 and the oldest patient diagnosed at 83 years old. None of these patients had metastatic disease at the time of diagnosis (as per inclusion criteria). The oncological treatment provided to these patients was varied, with a combination of surgery (33.3% receiving mastectomy and 66.7% receiving lumpectomy), chemotherapy (21.7%), radiation (76.7%), and endocrine therapy (80.0%). Of note, 20 patients received aromatase inhibitors (33.3%) as part of their cancer treatment, meaning that they require further screening as part of their follow-up care with BMD and lipid assessment. Interestingly, none of the patients included in our study received HER2-targeted therapy.

**Figure 1.**
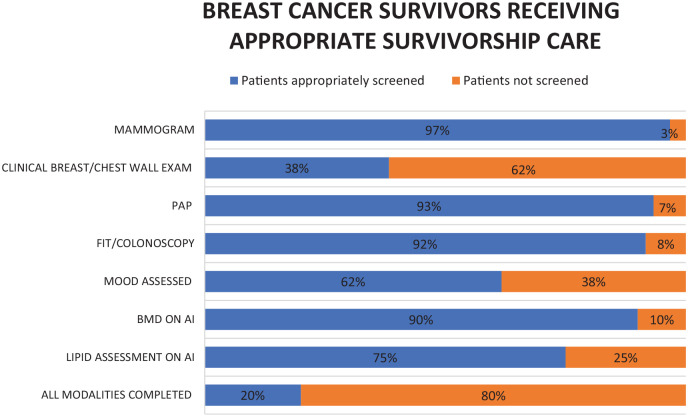
Percentage of breast cancer survivors receiving appropriate follow-up care by their PCP. *N* = 60.

The number of breast cancer survivors that received care concordant with all guidelines was 12 out of 60 (20.0%) (Figure 1). Most patients received an annual mammogram (58 out of 60, 96.7%). Chest/breast wall exams were performed in 23 out of 60 (38.3%) patients and 37 out of 60 (61.7%) were screened appropriately for psychosocial distress. Of the 20 patients who received aromatase inhibitors, 18 had BMD assessments every 2 years (90.0%) and 15 had lipids assessed annually (75.0%). Screening obtained was strongly concordant with preventative care guidelines, as 56 out of 60 (93.3%) received a Pap test every 3 years (or cervical cancer screening was not indicated as patient was past the age requirement or had a previous hysterectomy), and 55 out of 60 patients (92.0%) received timely colon cancer screening with either FIT or screening colonoscopy (Table 2).

**Table 2.** Completion of recommended survivorship care. *N* = 60 (for all except patients requiring lipids and BMD assessment, *N* = 20).

Recommended Survivorship Care	Completed	Not completed
Cancer surveillance		
Mammograms	58 (96.7%)	2 (3.3%)
Clinical breast exams	23 (38.3%)	37 (61.7%)
Preventative care		
FIT	55 (91.7%)	5 (8.3%%)
Pap	56 (93.3%)	4 (6.7%)
Management side effects		
Mood assessment	37 (61.7%)	23 (38.3%)
Lipids	15 (75.0%)	5 (25.0%)
BMD	18 (90.0%)	2 (10.0%)
All modalities	12 (20.0%)	48 (80.0%)

BMD: bone mineral density; FIT: fecal immunohistochemistry testing.

The age-specific categories contained 7 (age <50), 13 (50–59), 22 (60–69), and 18 (⩾70) patients. Overall, breast cancer survivorship follow-up was not significantly different across the age groups (*p* = 0.9) in any of the modalities. The percentage of patients who received annual mammograms across all age categories was similar with >85% concordance with guidelines. Assessment of mood and clinical breast exams were done with limited guideline concordance across the different age groups. High rates of Pap tests and colon cancer screening were noted across each age group. Age stratification was not analyzed for BMD and lipid assessment due to the small sample size of patients on aromatase inhibitors (20 patients total) (Table 3).

**Table 3.** Completion of recommended survivorship care stratified by age. *N* = 60.

Age	Mammograms (%)	Pap (%)	Mood assessment (%)	Clinical breast exams (%)	FIT (%)
<50	85.7	100.0	57.1	28.6	N/A
51–59	100.0	84.6	69.2	46.1	84.6
60–69	100.0	90.9	72.7	45.4	95.5
⩾70	94.4	N/A	44.4	27.8	88.9

FIT: fecal immunohistochemistry testing.

**Figure 2.**
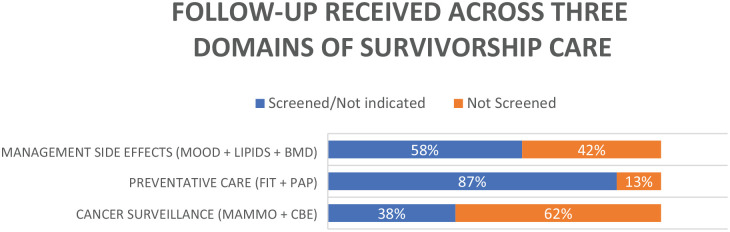
Comparison of guideline concordance of the three domains of breast cancer survivorship care.

In terms of the 3 domains of survivorship care, cancer surveillance, which is comprised of annual mammograms and routine clinical breast examinations, was completed appropriately in 23 out of 60 patients (38.3%). Management of side effects, including assessment of mood, lipids, and BMD was completed in 35 out of 60 patients (58.3%). Preventative care, including screening for cervical and colon cancer, was completed in 52 out of 60 patients (86.7%). The quality of breast cancer survivorship care received was significantly different depending on the domain assessed (*p* < 0.01) (Figure 2) and was completed across all domains in 20% of cases (Figure 1).

## Discussion

Breast cancer survivors cared for by PCPs at a large, academic family medicine teaching practice had poor overall concordance with survivorship follow-up guidelines. Only 20% of breast cancer survivors received appropriate care across all domains. PCPs were effective at completing mammograms, lipids, and BMDs, as well as colon and cervical cancer screening; however, mood assessments and clinical breast exams were not routinely performed. The likelihood that follow-up care is concordant with guidelines is not dependent on the patient’s age at diagnosis but significantly differs across each of the three domains of care. This study highlights specific care gaps that could be targeted in efforts to improve healthcare for breast cancer survivors after they are discharged from oncology.

Our results are consistent with similar analyses which have shown that only 46.4% of PCPs in a southeastern Ontario community were routinely following breast cancer survivorship guidelines and that just over one-quarter of PCPs were aware of all the recommendations for survivorship care.^6^ This study had similar findings of higher rates of annual mammograms (87.8%), but poorer compliance in areas such as screening for distress (14.6%) and fatigue (11.0%). These results indicate a consistent lack of education for PCPs around specific survivorship follow-up requirements. This is relevant as several systematic reviews have shown that breast cancer survivors have a higher prevalence of symptoms of depression than unaffected adult females and these symptoms tend to be persistent for greater than 5 years after diagnosis.^10,11^

We note that mammograms, Pap, and FIT/screening colonoscopies all have excellent guideline concordance across all age categories. This finding likely reflects automated reminders that are be placed in EMRs which prompt screening activities. These reminders can be customized to differing intervals, such as yearly mammograms for breast cancer survivors. At the time of this study, there were also financial incentivizations for screening programs that could have improved PCP uptake of cancer screening.^12^ Financial incentives for cancer screening have been shown to create a modest increase in routine screening^13^ and may also improve screening in higher risk populations as well. Although WBC care plans were present for all patients, these care plans are passive scanned documents and do not initiate reminders for care. The high concordance noted for care when there are active reminders highlights the need for similar strategies across all aspects of survivorship care, such as mood and clinical breast exams.

There is debate about the utility of routine clinical breast examinations for the detection of cancer recurrences. In an institutional review from Dublin, Ireland, researchers found that 46% of breast cancer recurrences were detected clinically.^14^ Conversely, in a large retrospective review of the WBC Program in Ottawa, Canada, researchers found that routine clinic exams detected only 2 out of 206 (1.14%) breast cancer recurrences and new primaries in breast cancer survivors, with most recurrences identified by patient-reported symptoms and routine imaging.^15^ In our study, only 38.3% of patients received timely clinical breast exams in concordance with guidelines. These results were not dependent on age at diagnosis; however, at both extremes of age (under 50 and over 70) patients tended to have fewer breast exams. Given that recurrences may be identified by patient-reported symptoms, it is key to empower patients, giving them the resources and autonomy to self-survey for potential recurrence so that they can bring any findings to medical attention if they occur between scheduled appointments. Current survivorship management may not adequately support patients in this regard.

The need for increased support of the transition in care from cancer programs to PCPs has been previously highlighted, specifically, the need to support communication, include self-management strategies, and pivot to digital health technologies to help address system capacity issues.^16^ Indeed, researchers have established that standardized communication between specialists and PCPs through specialized cancer care plans alone were insufficient to ensure survivorship guideline concordance.^17^ The “Survivor Advisor” is a digital survivorship tool which is currently being piloted at the Ottawa Hospital. *Survivor Advisor* creates a customizable survivorship pathway built on the patient’s specific breast cancer treatment and sends patients reminders for key investigations, recurrence screens, and timely follow-up care.^18^ The presence of care gaps noted in this study, along with improved outcomes with automation, and the need for increased patient awareness of symptoms to prompt presentation of recurrences which occur between appointments, suggests that a digital solution which empowers patients may help to close the gaps. Empowering patients through support with digital survivorship tools may help to reduce time demands on busy clinicians and streamline survivorship care-specific appointments.

This study is limited by the small sample size of included charts, and the assessment of patients from one academic center only. Nevertheless, it reflects an academic FHT which trains family medicine residents, and so should demonstrate the highest standard of care. A power analysis for sample size calculation was not completed. Our study was limited by the lack of socioeconomic data, as this information was not available in the chart review. Socioeconomic factors may influence survivorship care, as breast cancer survivors who are older, black, less educated, and of lower socioeconomic status have been found to have lower rates of mammograms.^19^ Our study likely underestimates the evaluation of mood and anxiety in breast cancer survivors, as we can only account for what is formally documented in the EMR. Finally, beyond depression and anxiety, it is not clear from our chart review if PCPs adequately addressed the full range of side effects that patients may be experiencing post-breast cancer therapy. Long-term impacts of breast cancer therapies may span from fear of recurrence to financial toxicity, sexual and cognitive dysfunction, and physical issues such as lymphedema, endometrial cancer, congestive heart failure, neuropathy, and pain, to name a few.^20^ The management of these side effects are becoming increasingly complex with ongoing treatment advances, as agents such as immunotherapy and CDK4/6 inhibitors are now integrated into the adjuvant setting.^21,22^ These important aspects of breast cancer survivorship care were beyond the scope of this retrospective review and are referenced in further detail in the Breast Cancer Survivorship Tool.^8^ The deficiencies in care highlighted in this study likely reflect only the tip of the iceberg and the educational gap for PCPs around survivorship care will continue to widen without ongoing adequate supports.

## Conclusion

This study provides data on the effectiveness of PCP care of breast cancer survivors, which is important given that care of these patients is increasingly occurring exclusively in the primary care domain. We show that PCPs are only providing care concordant with guidelines 20% of the time. PCPs were highly effective at preventative care, but survivorship-specific care was suboptimal. This data highlights key care gaps within our current survivorship care delivery model and underlines the need for novel strategies, such as digital tools, which allow for active, automated prompts and patient education and empowerment to optimize care and subsequent outcomes.

## Declarations

**Ethics approval and consent to participate:** This study was designed for institutional quality improvement and therefore ethics approval was waived. Based on the “Is your Project Research or QI?” guideline and checklist from OHSN-REB, and after consultation with the Department of Family Medicine’s FMRSP reviewers, the project was classified as QI. It falls under TCPS 2, Article 2.5, which states that QI activities, program evaluations, and performance reviews for internal purposes do not constitute research and are exempt from REB review. As such, formal ethics approval was not sought. Consent to participate in this study was not required as this manuscript does not contain individual-level information such as personal medical information or images and is solely for the purposes of quality improvement.

**Consent for publication:** Not applicable.

**Availability of data and materials:** Not applicable.
